# Chronic Distal Biceps Tendon Rupture: A Case Report of Single-Incision Repair With Tightrope and Flexor Carpi Radialis Autograft Augmentation

**DOI:** 10.7759/cureus.60663

**Published:** 2024-05-20

**Authors:** Abhiram Kannan, Aebel Raju, Ayyappan V Nair, Yonsik Yoo, Azaid Sait, Jimmy J Meleppuram, Prince Shanavas Khan

**Affiliations:** 1 Orthopedics and Traumatology, Aster Malabar Institute of Medical Sciences (MIMS), Calicut, IND; 2 Orthopedics and Traumatology, Apollo Adlux Hospital, Kochi, IND; 3 Orthopedics and Traumatology, Manipal Hospital Whitefield, Bangalore, IND; 4 Orthopedic Surgery, Hallym University Chuncheon Sacred Heart Hospital, Chuncheon, KOR; 5 Orthopedics, Aster Malabar Institute of Medical Sciences (MIMS), Kozhikode, IND; 6 Orthopedics and Traumatology, Apollo Adlux Hospital, Kozhikode, IND; 7 Orthopedics and Traumatology, Apollo Adlux Hospital, Ernakulam, IND

**Keywords:** sports injury, fcr augmentation, biceps augmentation, chronic biceps tear, distal biceps

## Abstract

A 48-year-old male presented with weakness in right upper limb flexion and supination three months post-road traffic accident and was diagnosed with a complete distal bicep tendon rupture. Urgent single-incision surgical repair augmented with the flexor carpi radialis tendon was performed using the tightrope reconstruction method for stability. This case underscores the importance of prompt recognition and intervention for distal biceps tendon tears to prevent long-term functional impairment, emphasizing the critical role of surgical reattachment. Delayed medical care may compromise work capabilities and surgical success.

## Introduction

Distal biceps tendon injuries are infrequent (2.55 per 100,000 patient-years) and usually arise from a specific combination of movements, such as when the elbow extends. At the same time, the biceps muscle is being flexed, often occurring during heavy lifting [1]. This action places excessive strain on the tendon, surpassing its natural limits and detrimentally affecting the ability to both flex the elbow and supinate. Careful consideration of various factors like strength, pain levels, the individual’s age, and the extent of tendon damage all play pivotal roles in determining the best course of action. The management approach spans a spectrum, ranging from conservative methods to definitive surgical intervention. Notably, the report highlights a crucial aspect: in cases where a complete tear of the biceps tendon remains untreated, the ability to flex the elbow might still be retained to some degree due to assistance from the brachialis muscle. However, supination strength experiences a substantial drop of approximately 50% [2]. Such a rupture also negatively impacts endurance and the performance of tasks that necessitate a firm grip. Beyond physical discomfort, it outlines how this type of injury can hinder day-to-day activities involving grooming and the use of handheld manual tools [2].

## Case presentation

**Figure 1:**
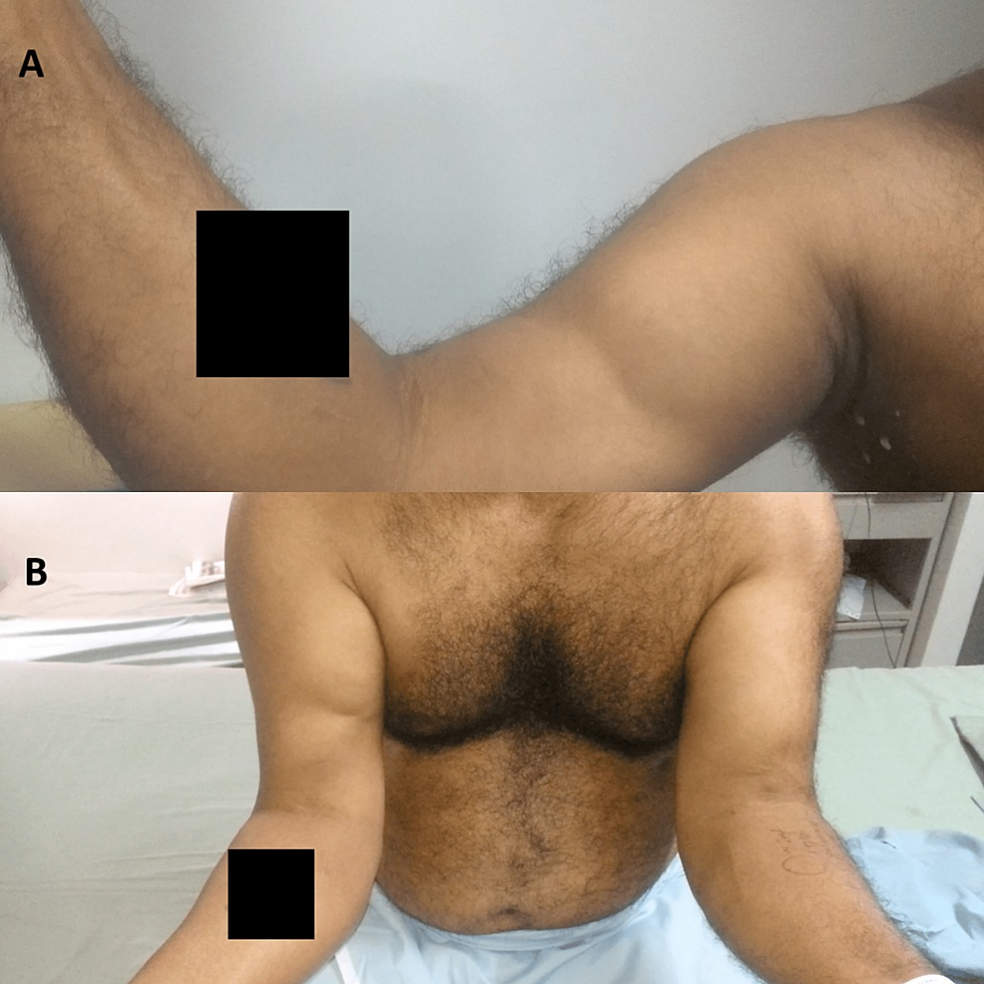
(A) Reverse Popeye sign. (B) Comparison of the distal biceps tendon rupture in the right arm with the normal left arm

**Figure 2:**
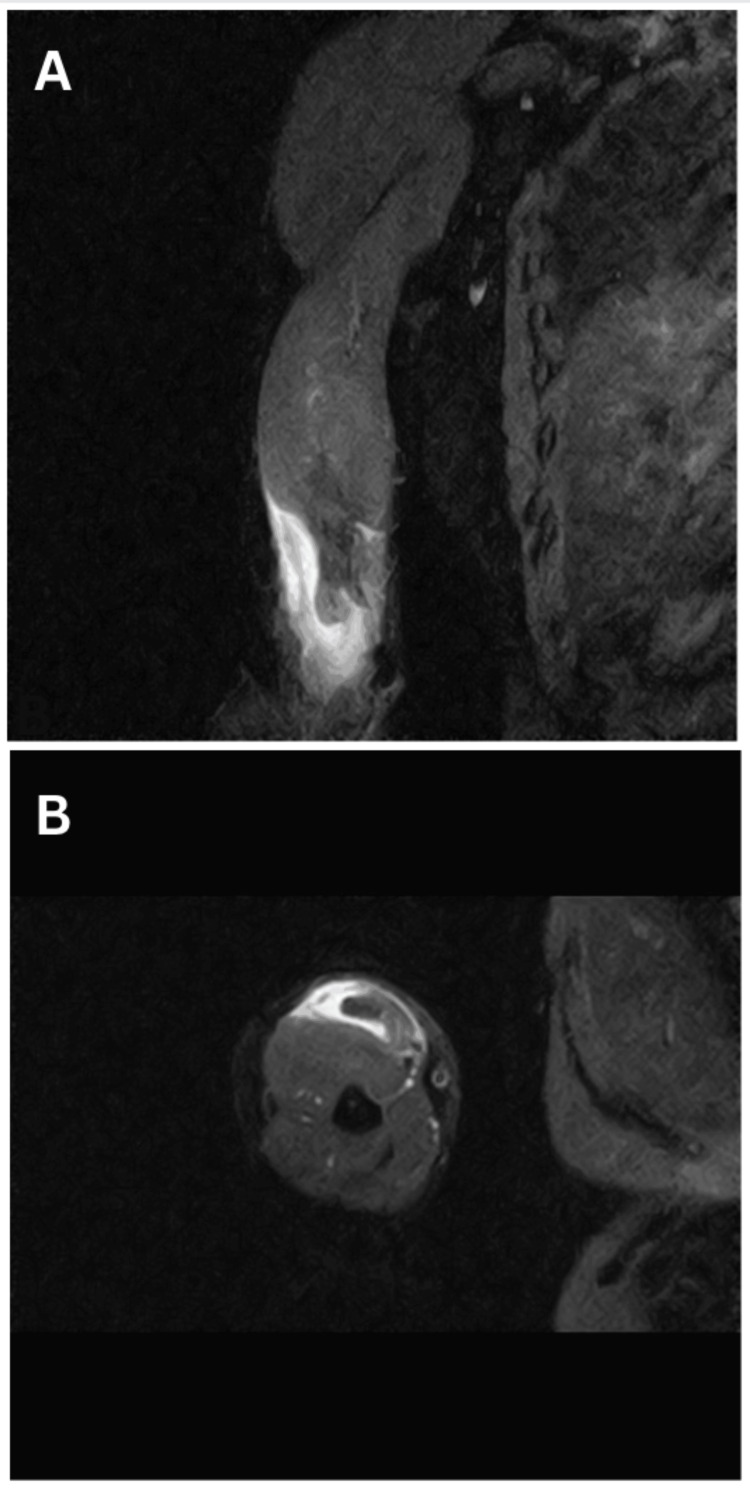
(A) Sagittal MRI image showing distal biceps tendon rupture. (B) Axial MRI image showing distal biceps tendon rupture

A 48-year-old male who had been involved in a road traffic accident experienced persistent right upper limb pain, with his dominant hand affected. The initial X-ray showed no injuries. After three months, he presented with increased muscle bulk and ongoing weakness, prompting an evaluation at our center. Upon clinical examination, we observed a positive reverse Popeye sign (Figure 1A, 1B), a negative biceps squeeze, and a hook test indicative of complete distal biceps tendon rupture, resulting in grade 4 weakness in supination and flexion. Radiological assessment using MRI confirmed a complete distal tendon rupture with retraction (Figure 2).

**Figure 3:**
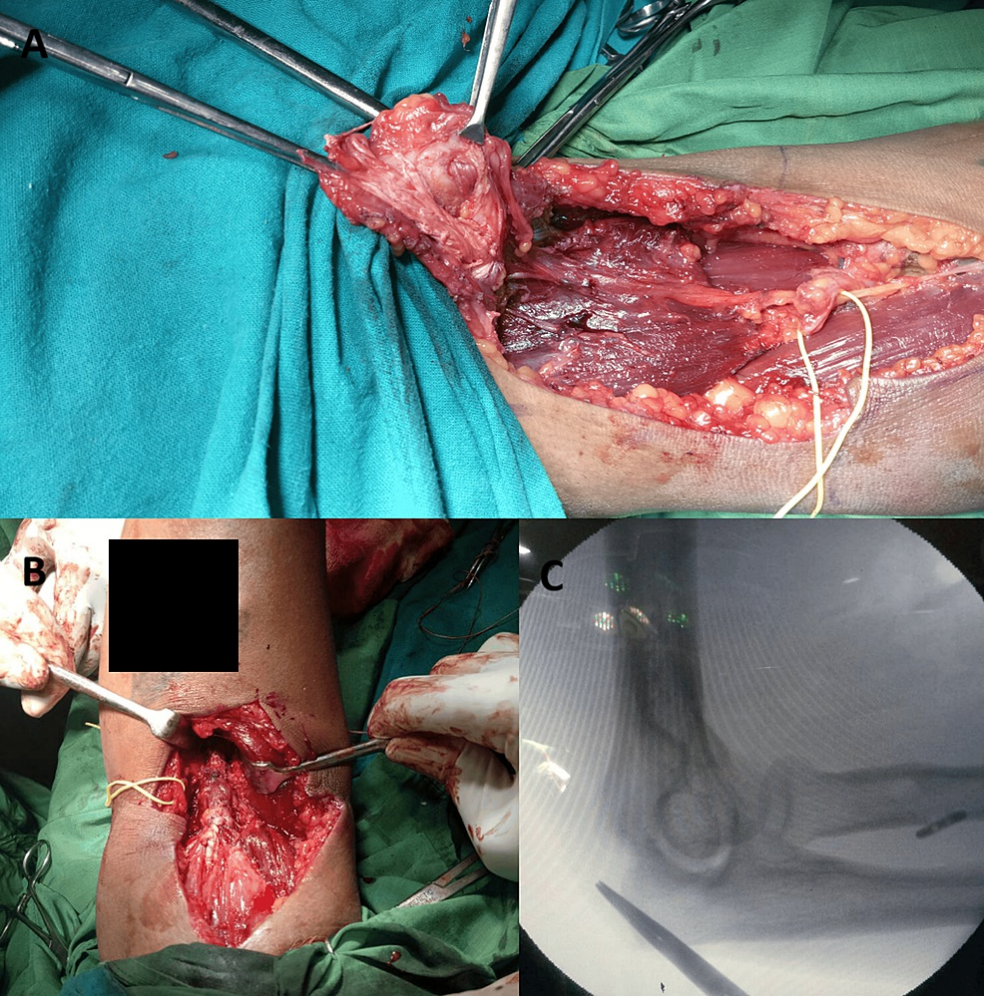
(A) Intraoperative image showing distal biceps rupture. (B) Distal biceps repair performed with autograft augmentation. (C) Image of the arm showing tightrope attachment

The patient underwent an open anterior distal biceps tendon repair using a modified Henry’s approach. A 12-cm incision provided access to the biceps tendon and radial tuberosity. Safeguarding vital structures like the median nerve, brachial artery, and lateral antebrachial cutaneous nerve was a priority during dissection. The biceps tendon had retracted 8 cm with fraying of the distal ends (Figure 3A). A cable grafting technique was employed, utilizing the ipsilateral flexor carpi radialis, reinforced with fiber tape, to bridge the gap and ensure tension (Figure 3B, 3C).

Following the surgery, the patient’s elbow was immobilized at an angle of 60 degrees of flexion using a plaster of Paris slab. Subsequently, a range of motion (ROM) elbow brace was applied, featuring an extension stopper. This brace allowed controlled and gradual increases in the ROM to prevent the tendon from pulling out. During the postoperative period, activities that could potentially apply excessive force to the repaired tendon, such as forceful supination and weight loading, were avoided.

**Figure 4:**
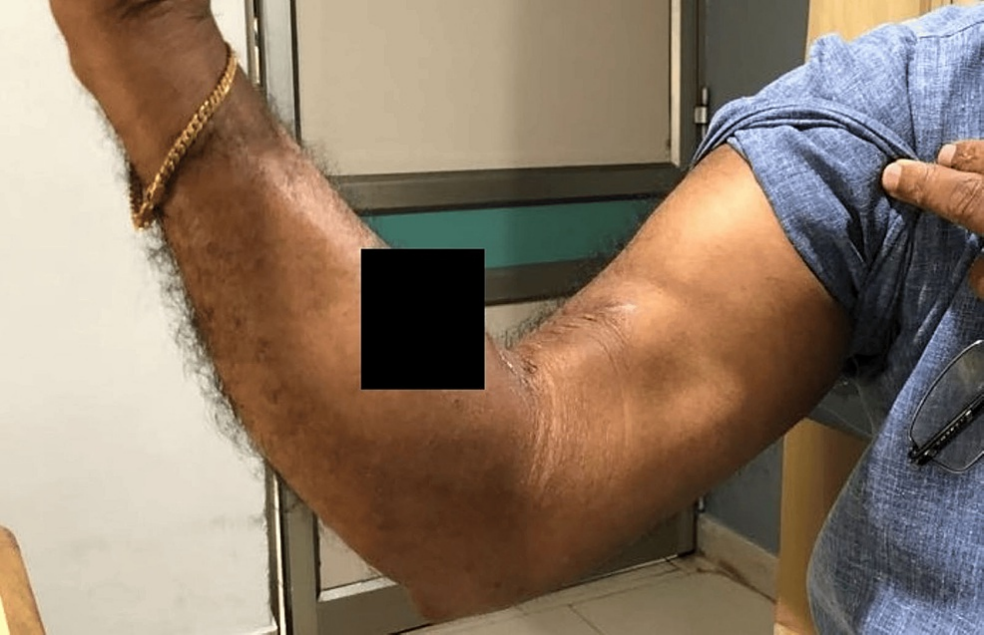
Postoperative image at six-month follow-up

Around six weeks post-surgery, resistance exercises were initiated, and the patient regained full ROM in the affected arm. At the six-month, one-year, and five-year follow-up marks, the patient demonstrated grade 5 biceps power (Figure 4).

## Discussion

When choosing a nonoperative treatment pathway, it is essential to consider the primary role of the biceps muscle in supination and its secondary involvement in flexion. In such cases, it is important to anticipate a significant reduction in functional strength, with an approximate 50% decrement in supination capability, a 30% diminution in flexion capacity, and a 15% decline in grip strength [3].

The biceps tendon, with short and long heads, attaches uniquely to the radius - the short head (60 mm^2^) and the long head (48 mm^2^) - posteriorly or ulnarly. Surgical repair focuses on precise reattachment [4]. The distal biceps tendon’s insertion zone is divided into three significant vascular zones. The proximal segment relies on brachial artery branches; transitionally, the recurrent branch of the posterior interosseous artery plays a crucial role, with an avascular transitional region susceptible to injury. Full forearm pronation leads to impingement at the proximal radioulnar joint, elevating injury risk by nearly 50% [5,6]. The complex anatomy and biomechanical factors form the basis of distal biceps tendon injuries [4]. Precise biceps tendon reattachment is crucial for effective treatment, especially in challenging cases with persistent injuries or tough scar tissue. Grafts, like hamstring, flexor carpi radialis, palmaris longus, and fascia lata autografts, are well-explored options. Additionally, allografts such as the Achilles tendon are studied as credible alternatives for challenging cases [7].

The surgical approach has to be preplanned, with dual-incision techniques offering augmented exposure alongside heightened concerns regarding heterotopic ossification pathological processes characterized by anomalous bone formation within soft tissues. Conversely, the single-incision approach garners favor in many clinical scenarios owing to its potential to attenuate the risk of ossification, especially when juxtaposed against methodologies such as Morrey’s modified Boyd and Anderson’s double-incision technique. While the single-incision technique does carry an elevated likelihood of posterior interosseous nerve injury, employing hypersupination of the forearm emerges as a strategic mitigatory measure. Strategic adoption of endobuttons emerges as a discerning choice, not only due to its documented superior biomechanical robustness but also due to its demonstrated propensity for minimizing mechanical failure. Consideration of these factors augments the overall durability and resilience of the repair process [8].

The endoscopic repair method is gaining attention, offering cosmetic benefits despite challenges like brachial vessel injury and nerve issues, especially in altered cubital fossa anatomy. While effective for graft-free reconstructions with quick healing, it may be less suitable for tough scar tissue and not ideal for cases with dense scar tissue. Balancing surgery and cosmetic preferences provides a precise view but may not be as effective when grafts are required [9].

Postoperative rehabilitation spans six months, beginning with splinting and gradually progressing to extension restoration. Two weeks of immobilization was done. During the three- to six-week period, activities ranged from flexion arcs of 60 to 120 degrees with assisted movements. At six weeks, incorporating resistance exercises becomes pivotal, catalyzing methodical muscular strength restoration, culminating in unrestricted activity endorsement at six months [10].

## Conclusions

Distal biceps injuries, a rarity in orthopedic clinical practice, require a tailored decision-making approach to optimize functional outcomes for patients. The success of outcomes is contingent upon considerations such as heterotopic ossification and neurovascular damage, necessitating careful evaluation of benefits on a case-by-case basis, especially in chronic injury scenarios. In instances of chronic retracted tears, the discussion further encompasses the imperative consideration of graft utilization to enhance tissue repair and restore optimal function.
